# Implications of monsoon season and UVB radiation for COVID-19 in India

**DOI:** 10.1038/s41598-021-82443-6

**Published:** 2021-02-02

**Authors:** Rahul Kalippurayil Moozhipurath, Lennart Kraft

**Affiliations:** Faculty of Economics and Business, Goethe University Frankfurt, Theodor-W.-Adorno-Platz 4, 60629 Frankfurt, Germany

**Keywords:** Immunology, Ecology, Environmental sciences, Environmental social sciences, Diseases, Health care, Medical research, Pathogenesis, Risk factors

## Abstract

India has recorded 142,186 deaths over 36 administrative regions placing India third in the world after the US and Brazil for COVID-19 deaths as of 12 December 2020. Studies indicate that south-west monsoon season plays a role in the dynamics of contagious diseases, which tend to peak post-monsoon season. Recent studies show that vitamin D and its primary source Ultraviolet-B (UVB) radiation may play a protective role in mitigating COVID-19 deaths. However, the combined roles of the monsoon season and UVB radiation in COVID-19 in India remain still unclear. In this observational study, we empirically study the respective roles of monsoon season and UVB radiation, whilst further exploring, whether the monsoon season negatively impacts the protective role of UVB radiation in COVID-19 deaths in India. We use a log-linear Mundlak model to a panel dataset of 36 administrative regions in India from 14 March 2020–19 November 2020 (n = 6751). We use the cumulative COVID-19 deaths as the dependent variable. We isolate the association of monsoon season and UVB radiation as measured by Ultraviolet Index (UVI) from other confounding time-constant and time-varying region-specific factors. After controlling for various confounding factors, we observe that a unit increase in UVI and the monsoon season are separately associated with 1.2 percentage points and 7.5 percentage points decline in growth rates of COVID-19 deaths in the long run. These associations translate into substantial relative changes. For example, a permanent unit increase of UVI is associated with a decrease of growth rates of COVID-19 deaths by 33% (= − 1.2 percentage points) However, the monsoon season, mitigates the protective role of UVI by 77% (0.92 percentage points). Our results indicate a protective role of UVB radiation in mitigating COVID-19 deaths in India. Furthermore, we find evidence that the monsoon season is associated with a significant reduction in the protective role of UVB radiation. Our study outlines the roles of the monsoon season and UVB radiation in COVID-19 in India and supports health-related policy decision making in India.

## Introduction

COVID-19 has caused unparalleled economic and health disruptions in India, the second most populated country in the world with over 1.3 billion people. As of 12 December 2020, India has reported 142,186 deaths COVID-19 deaths across 36 administrative regions, placing India third in the world behind the US and Brazil^1^.

Prior studies indicate that south-west monsoon season (monsoon season) plays a role in the dynamics of contagious diseases, which tend to peak post-monsoon season^2^. A sudden increase in contagious diseases during and post-monsoon season may stress India’s healthcare system^3,4^. Recent observational and clinical studies show that vitamin D deficiency might be linked to incidence^5–8^, severity^7,9^ and mortality^10–12^ associated with COVID-19^13^. Emerging studies also show that vitamin D and its primary source Ultraviolet-B radiation (UVB) may play a protective role in mitigating COVID-19 deaths^14^. Limited hours of sunlight and dense cloud cover^15^ limit the intensity of UVB radiation, mitigating its protective role^14^ during the monsoon season. Further, the onset of the monsoon season may also alter the behaviour of people limiting their exposure to UVB radiation. Despite the importance of the monsoon season and the UVB radiation, their respective roles in COVID-19 in India are still unclear. To the best of our knowledge, so far, no empirical study has explored these roles of the monsoon season and UVB radiation in COVID-19 deaths in India.

In this observational study, we empirically describe the roles of the monsoon season, UVB radiation and further explore, whether the monsoon season is associated with a reduction in the protective role of UVB radiation in COVID-19 deaths in India. After controlling for various confounding factors, we observe that in the long run a unit increase in UVI and the monsoon season are separately associated with 1.2 percentage points (p < 0.01) and 7.5 percentage points (p < 0.05) decline in COVID-19 deaths growth rate. However, we find evidence that in the long run, the monsoon season is associated with a reduction in the protective role of UVB radiation by 0.92 percentage points (p < 0.01).

## Impact of monsoon on healthcare system, UVB radiation and COVID-19 deaths in India

Respiratory infections, such as those caused by influenza virus^16^ and human seasonal coronaviruses^17^, show geographic variation in terms of seasonality. For instance, influenza and human seasonal coronaviruses show a higher prevalence and a clear seasonality during winter months in higher latitudes; however, this seasonality tends to be limited in regions closer to equator^16,17^. Unlike regions in higher latitudes, studies indicate that in India the monsoon season and post-monsoon season may be associated with the peaks of contagious diseases like influenza, i.e., July–September^2,18,19^.

The onset of the monsoon season in India is associated with a change in the average levels of weather factors such as precipitation, cloud temperature, humidity and the UV Index (Ultraviolet Index). Studies show that weather factors such as humidity^20^, temperature^21^ and precipitation^21,22^ play a role in viral transmission (e.g., influenza). Recent studies also indicate that temperature and humidity may play a role in COVID-19 transmission^22,23^.

Prior studies indicate that heavy rainfall linked to the monsoon season may create situations favourable for the outbreaks of infectious diseases such as diarrheal disease, cholera, dengue, typhoid as well as respiratory diseases^24^. The consequences of possible coinfection with these infectious diseases and SARS-CoV-2 (severe acute respiratory syndrome coronavirus 2) are largely unknown^3,4^. The temporal overlap between these contagious diseases and COVID-19 may give rise to significant health care challenges^3^ during the monsoon season. Moreover, we anticipate this sudden increase in contagious diseases during the monsoon season may create stress in the healthcare system, further restricting the hospital capacity required for COVID-19 patients^3,4^. Additionally, heavy precipitation associated with the monsoon season may also cause traffic disruptions, limiting the transportation possibilities of COVID-19 patients, increasing the likelihood of COVID-19 deaths^4^.

Further, in addition to the above consequences in the healthcare system, another important consequence of the monsoon season is the higher precipitation and the reduced likelihood of UVB exposure and subsequently lower vitamin D levels^25^. Studies indicate that UV radiation inactivates viruses in fomite transmission^26^. UVB also plays another protective role via its role in vitamin D skin synthesis^27–31^, as dietary intake (natural food, fortified food or supplements) are usually insufficient^32^. Even in a country like India with plenty of sunshine, vitamin D deficiency is common due to reduced skin exposure and specific dietary habits such as vegetarianism^33^. UVB radiation and the likelihood of skin exposure & skin synthesis vary substantially depending on several factors such as seasons^32^, time^32^, latitude^32^, altitude^32^, active lifestyle^34,35^, dietary habits^32,36^, food fortification^32^, age^32^ and skin colour^32^. Prior studies indicate that most of Indians (Location: 8.4° N and 37.6° N^37^) belong to skin type V^37^ and the time required for recommended vitamin D synthesis for skin type V is 10–15 min at solar noon at 11.5° N throughout the year^37^. However, this period increases to 10–45 min with more duration in winter season at 29° N.

Early studies show a protective role of UVB and vitamin D in COVID-19^5,6,11,14^. 1,25-dihydroxyvitamin D (1,25 (OH)_2_D), an active form of vitamin D, plays a critical role in the modulation of innate as well as adaptive immune systems^38,39^, renin-angiotensin system (RAS)^39–41^ as well as in the modulation of the inflammatory response, reducing cytokine storm risk ^38,39^. It also stimulates antimicrobial peptides such as defensins and human cathelicidin^38,39,42,43^ with antiviral properties. Recent studies related to COVID-19 also indicate that vitamin D deficiency might be a risk factor not only for incidence^5–8^ but also for severity^7,9^ and mortality^10–12^ associated with COVID-19^13^.

Furthermore, the onset of the monsoon season may also alter the behaviour of people plausibly affecting the transmission of the virus and the immunity of the population. For instance, monsoon may affect the mobility of the people and thereby affecting the likelihood of transmission of the virus. Even though there are some sporadic days or even some hours of sunshine during the monsoon season, behavioural changes such as limited mobility and changes in dressing style associated with the monsoon season may affect the likelihood of exposure to UVB radiation. Similarly, changes in dietary patterns (e.g., limited availability of fish due to restricted fishing activities) associated with the monsoon season may also affect the immunity of the population.

In sum, the roles of the monsoon season and the UVB radiation in COVID-19 deaths in India are largely unknown. First, we anticipate that a sudden increase in contagious diseases along with COVID-19 during the monsoon season may create stress in the healthcare system and may increase the likelihood of COVID-19 deaths. Second, we expect that the monsoon season may affect the transmission likelihood of SARS-CoV-2 virus due to changes in the behaviour of people (e.g., restricted mobility of the population)^44^. Third, we anticipate that the likelihood of UVB radiation exposure declines substantially during the monsoon season, primarily due to lower sunshine hours, thick cloud cover and changes in the behaviour of people. Lower vitamin D levels due to the reduced likelihood of UVB radiation exposure may lead to lower immunity and increased mortality rate^14,32^ during the monsoon season. Therefore, in light of the emerging evidence concerning vitamin D and COVID-19, we aim to explore the role of monsoon season, UVB radiation and how monsoon season potentially mitigates the protective role of UVB radiation in COVID-19.

## Methods

### Description of data

In order to identify the association of UVI, monsoon season, and their interaction with COVID-19 deaths, we constructed the dataset outlined in Table 1. We collected COVID-19 data across 36 administrative regions (28 states and eight union territories) in India, covering 251 days from 14 March 2020 until 19 November 2020. Thirty-five of these administrative regions reported more than 20 COVID-19 infections on 19 November 2020. We focus on those 35 administrative regions to ensure that our results are not biased by those regions that are at a very early stage of the COVID-19 outbreak. Further, we drop the first 20 daily observations of each administrative region after the reporting of the first COVID-19 infection. Thus, we ensure that our results are not biased by observations that at the very early stage of the outbreak.

**Table 1:** Summary of the dataset.

**Number of administrative regions of India in our data set**	36
> 0 cumulative number of COVID-19 deaths before 19 November 2020	35
> 20 cumulative number of COVID-19 infections before 19 November 2020	35
Time-period covered	14 March 2020–19 November 2020 (251 days)
Granularity of data	Daily
COVID-19 data source	https://api.covid19india.org/documentation/csv/
Latitude and longitude data source for each administrative region that is used to match weather data	Geocoder (Python)
Weather data source	https://darksky.net/
Monsoon season data source	https://indianexpress.com/article/india/from-june-2020-revised-monsoon-calendar-for-india-6364258/ https://mausam.imd.gov.in/imd_latest/contents/monsoon.php

The corresponding dataset consist of the cumulative number of daily COVID-19 deaths at an administrative region level. They also consist of the time of arrival of monsoon season for each administrative region, the daily ultraviolet index (UVI), an indicator of daily UVB radiation, as well as a set of additional daily weather parameters as control variables. These additional weather parameters include cloud index, stratospheric ozone level, visibility level, humidity level, minimum and maximum temperature. We source COVID-19 data from COVID19India.org and the weather data from darksky.net based on the latitude and longitude information of administrative regions that are provided by Geocoder, a geocoding library in Python.

We show the descriptive statistics of the dataset in Table 2. As of 19 November 2020, the cumulative number of daily COVID-19 deaths (cumulative COVID-19 deaths) for these 35 administrative regions were on average 3,800. The growth rate of the cumulative number of daily COVID-19 deaths (daily growth rates of COVID-19 deaths) across all administrative regions on 19 November was on average 0.53%. The average daily growth rate of COVID-19 deaths across all administrative regions and time was 3.6%. On average, the first reported COVID-19 infection in each administrative region happened 240 days before 19 November 2020. The average UVI across all regions and study period equals 7.9.

**Table 2:** Descriptive statistics of the data set.

Variable	Number of administrative regions	Number of observations	Mean	Std. dev	Min	Max
Cumulative COVID-19 deaths on 19 November 2020	35	35	3800	8100	2	46,000
Growth rate of cumulative COVID-19 deaths on 19 November 2020	35	35	0.0053	0.0060	0	0.026
Daily growth rate of cumulative COVID-19 deaths	35	6751	0.036	0.11	− 0.5	3
Time-passed by since first reported infection until 19 November 2020	35	35	240	18	179	251
Daily Ultraviolet index (UVI)	35	7728	7.9	2.4	3	15
Daily cloud index	35	7728	0.58	0.34	0	1.0
Daily stratospheric ozone level	35	7728	272	13	239	344
Daily visibility level	35	7728	15	1.7	1.0	16.1
Daily humidity level	35	7728	0.76	0.21	0.09	1.0
Minimum temperature per day within an administrative region	35	7728	21	6.8	− 16	32
Maximum temperature per day within an administrative region	35	7728	30	7.1	− 4.2	46

**Figure 1:**
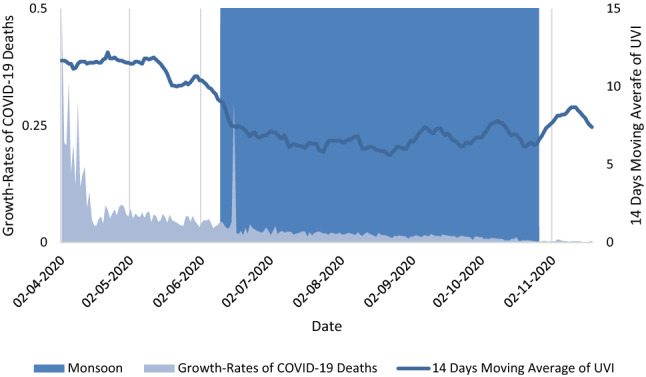
Illustration of the daily growth rates of COVID-19 deaths, Ultraviolet Index (UVI) and the monsoon season in Maharashtra, an administrative region in India.

Figure 1 illustrates the primary variables, i.e., the daily growth rates of COVID-19 deaths, 14 days moving average for UVI and the monsoon season for Maharashtra, an administrative region in India. Figure 1 shows a decline in the daily growth rates of COVID-19 deaths in Maharashtra over the study period. UVI in Maharashtra increases during the summer season, declines during the monsoon season and again increases after the withdrawal of the monsoon.

### Description of methodology

We use a Mundlak error correction model to estimate the association of UVI, monsoon season, and their interaction with the daily growth rates of COVID-19 deaths. We use 56 days moving average of the monsoon season, a dummy variable that indicates whether a specific day in an administrative region belongs to the monsoon season. Further, we use 56 days moving averages of the UVI and the interaction of this UVI variable with the monsoon season. The corresponding regression coefficients represent the associations of UVI and monsoon season with the daily growth rates of COVID-19 deaths. The coefficient of the interaction of UVI and the monsoon season represents the moderating effect of the monsoon season on the association of the UVI with the daily growth rates of COVID-19 deaths.

Our methodology controls for all time-constant confounding factors as well as various time-varying confounding factors such as region-specific time-trends and time-varying weather parameters. First, our model isolates all weather parameters from region-specific time-constant factors via Mundlak error correction model. This Mundlak model combines the robustness of a fixed-effects model and the efficiency of a random-effects model. Instead of demeaning the structural model to isolate the weather parameters analytically from region-specific time-constant factors, Mundlak model isolates those region-specific time-constant factors through the available weather parameters. Second, we include additional weather parameters in our analysis to isolate the association of UVI and monsoon from potentially confounding time-varying weather parameters such as cloud level, stratospheric ozone level, visibility level, humidity level, and minimum and maximum temperature. Third, we also control for further time-varying confounding factors by flexibly controlling for linear and quadratic region-specific time-trends. We describe our methodology and the interpretation of the estimated associations in more detail in Sects. 1 and 2 in the Supplementary Material.

## Results

We outline our main results in Table 3. Models 1 through 4 progressively include the variables—UVI, monsoon season, UVI and monsoon season as well as the interaction of UVI and monsoon season—in a step-wise manner. The coefficient capturing the interaction of UVI and the monsoon season represents the moderating effect of monsoon on the association of UVI with the daily growth rates of COVID-19 deaths. Model 4, our primary model that includes all of these variables, provides evidence of substantial and significant associations of UVI, monsoon season, and their interaction with COVID-19 deaths.

**Table 3:** Effect of the monsoon season, ultraviolet index (UVI) and their interaction on the cumulative COVID-19 deaths.

	Model 1	Model 2	Model 3	Model 4
	COVID-19 deaths	COVID-19 deaths	COVID-19 deaths	COVID-19 deaths
**Dependent variable**				
UVI	− 0.013** (− 2.84)		− 0.013** (− 2.76)	− 0.012** (− 2.64)
Monsoon		0.0073 (0.69)	0.0027 (0.25)	− 0.075* (− 2.33)
UVI × monsoon				0.0092** (2.56)

Time trend of growth rate	Linear and square (region-specific)	Linear and square (region-specific)	Linear and square (region-specific)	Linear and square (region-specific)
**Control variables**				
Cloud index	Yes	Yes	Yes	Yes
Stratospheric ozone level	Yes	Yes	Yes	Yes
Visibility level	Yes	Yes	Yes	Yes
Humidity level	Yes	Yes	Yes	Yes
Temperature (min and max)	Yes	Yes	Yes	Yes
Number of estimates	7 (+ 7 MEC + 70 RSTE)	7 (+ 7 MEC + 70 RSTE)	8 (+ 8 MEC + 70 RSTE)	9 (+ 9 MEC + 70 RSTE)
Number of observations	6751	6751	6751	6751
Number of regions	35	35	35	35
R-squared within	10.26%	10.16%	10.26%	10.34%

t-statistics that tests the statistical significance (two-sided t-test) of the estimated associations between the dependent variable and the respective independent variable are in parentheses. MEC represents Mundlak error correction estimates which isolate the weather variables from region-specific time-constant factors. RSTE represents region-specific time-effects.

*p < 0.05, **p < 0.01.

We find that a permanent unit increase of UVI is associated with a decline of 1.2 percentage points (p < 0.01) in the daily growth rates of COVID-19 deaths. The monsoon season is associated with a decline of 7.5 percentage points (p < 0.05) in the daily growth rates of COVID-19 deaths. However, the monsoon season also mitigates the protective role of UVB radiation in reducing the daily growth rates of COVID-19 deaths by 0.92 percentage points (p < 0.01). These associations translate into substantial relative changes in the daily growth rates of COVID-19 deaths. For example, a permanent unit increase of UVI is associated with a decline in the daily growth rates of COVID-19 deaths by 33% (= − 1.2/3.6) relative to the average daily growth rates of COVID-19 deaths. However, the monsoon season mitigates this protective role of UVI by 77% (= 0.92/ − 1.2). Models 1 through 3 of Table 3 show a stable association of UVI with the daily growth-rates of COVID-19 deaths. Monsoon season decreases the daily growth rates of COVID-19 deaths only after partialling out its mitigating effect on UVI.

Tables S4.1 and Table S4.2 in the Supplementary Material demonstrate that the results are consistent even after using different time windows (4–11 weeks) to construct the moving averages of weather parameters. Robustness checks in Table S5 in the Supplementary Material outline that our estimations are consistent even after using flexible time trends and different governmental measures.

**Figure 2:**
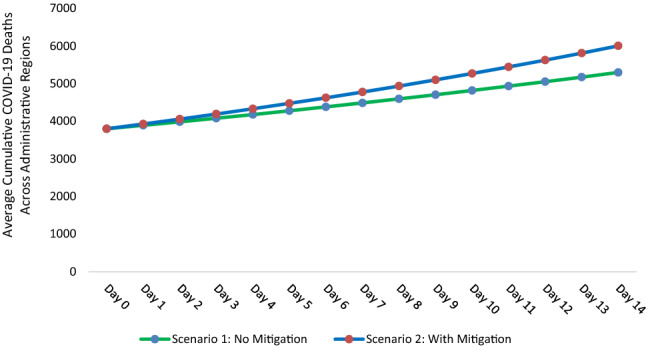
Comparison of scenario 1 (UVB radiation’s protective role is not mitigated by the monsoon season) vs. scenario 2 (UVB radiation’s protective role is mitigated by the monsoon season).

Figure 2 illustrates the mitigation effect of the monsoon season on the protective role of UVB radiation in COVID-19. We compare two scenarios (i) *scenario 1* Monsoon does not mitigate the protective role of UVB radiation; (ii) *scenario 2* Monsoon mitigates the protective role of UVB radiation. In scenario 1, we simulate the effect of a permanent unit increase of UVI on cumulative COVID-19 deaths, when the UVB radiation’s protective role is not mitigated by the monsoon season. In scenario 2, we simulate the same effect, when the UVB radiation’s protective role is mitigated by the monsoon season.

For this simulation, we use the average number of COVID-19 deaths at the end of the observational period, i.e., 3800, as cumulative COVID-19 deaths at day 0 (see Fig. 2). Further, we use the average daily growth rate of COVID-19 deaths (3.6%). In scenario 1, where the protective role of UVB radiation is not mitigated, we estimate that a permanent unit increase in UVI is associated with an average daily COVID-19 growth rate of 2.4% (3.6–1.2 p.p). Similarly, in scenario 2, where UVB radiation’s role is mitigated, we estimate that a permanent unit increase in UVI is associated with an average daily COVID-19 growth rate of 3.32% (3.6–1.2 p.p + 0.92 p.p). Figure 2 outlines that scenario 2 has 707 or 13% (707/5296) more COVID-19 deaths after 14 days compared to scenario 1.

## Discussion

Our empirical results outline that the monsoon season and UVB radiation are independently associated with a decline in the daily growth rate of COVID-19 deaths, thereby indicating their respective roles in India. However, the monsoon season is also associated with significant mitigation of this protective role of UVB radiation. Specifically, we find that a unit increase in UVI and the monsoon season are separately associated with 1.2% points, and 7.5% points decline in the daily growth rate of COVID-19 deaths in India in the long run. The monsoon season in India mitigates this protective effect of UVI by 0.92 percentage points, plausibly due to the limited likelihood of the UVB exposure. We find these results to be consistent across different model specifications. The protective role of UVB radiation may plausibly be due to its role in skin synthesis of vitamin D. These results are in line with the recent studies related to COVID-19 that indicate vitamin D deficiency might be a risk factor not only for incidence^5–8^ but also for severity^7,9^ and mortality^10–12^ associated with COVID-19^13^. However, we may not be able to exclude other protective roles of UVB radiation—for example via other mediators—nitric oxide^9,10^, cis-urocanic acid^27,45,46^ or via inactivation of viruses in fomite transmission^26^. Although we anticipate a sudden increase in contagious diseases along with COVID-19 during the monsoon season may increase the likelihood of COVID-19 deaths, the findings suggest that the monsoon season is associated with a decline in the daily growth rates of COVID-19 deaths. Future studies can explore this missing link between the monsoon season and the reduction in the daily growth rates of COVID-19.

In our analyses, we control for all time-constant region-specific factors and various time-varying confounding factors, such as region-specific time-trends and time-varying weather parameters^14^. However, we acknowledge that we may not be able to exclude other time-varying factors (e.g., varying travel patterns of infected individuals), which might bias our results^14^. Moreover, we also acknowledge that the results of our study cannot serve as health guidance for India. However, we hope our results prompt further clinical research in India specifically to establish the role of sensible sunlight exposure or vitamin D in mitigating COVID-19 deaths, especially during the monsoon season.

Establishing the effectiveness of sensible solar UVB radiation exposure or vitamin D supplementation via clinical studies could substantially advance the control of COVID-19 pandemic at scale in India. The results of these clinical studies can further guide policy decision making in India, especially during the monsoon season. This type of policy intervention would be desirable for India not only due to its lower risk and costs but also due to its scalability across India’s 1.3 billion people whose economic means vary significantly.

Further, we note that sensible sunlight exposure^14^ is important for human health, whereas disproportionate solar UV exposure may lead to health hazards such as aging^47^, wrinkles^47^, sunburn^32^ and DNA damage^47^. Specifically, disproportionate UVB exposure is associated with basal cell and squamous cell carcinoma^48^.

## Supplementary Information

Supplementary Information.

## Data Availability

The data used in the study are from publicly available sources. Data regarding COVID-19 are obtained on 20 November 2020 from https://api.covid19india.org/documentation/csv/. Data regarding weather is obtained from *Dark Sky* on the 20 November 2020 and can be accessed at https://darksky.net/. Latitude and longitude information is obtained via Geocoder (Python), whereas monsoon season data is obtained from https://indianexpress.com/article/india/from-june-2020-revised-monsoon-calendar-for-india-6364258/ as well as from https://mausam.imd.gov.in/imd_latest/contents/monsoon.php on the 20 November 2020. We will make specific data set used in this study available for any future research. Interested researchers can contact one of the authors via email to get access to the data.
