# Supplementary material for: Implications of monsoon season and UVB radiation for COVID-19 in India
Source: Sci Rep. 2021 Feb 2;11:2757. doi: 10.1038/s41598-021-82443-6 (PMC7854580; doi:10.1038/s41598-021-82443-6)
Supplement: Supplementary file 1 — Supplementary Information. [file 41598_2021_82443_MOESM1_ESM.pdf]

Implications of Monsoon Season & UVB Radiation for COVID-19 in India

Supplementary Material

Rahul Kalippurayil Moozhipurath*[1], Lennart Kraft[1]

(Faculty of Economics and Business, Goethe University Frankfurt[1])

Rahul Kalippurayil Moozhipurath; Lennart Kraft; Faculty of Economics and Business, Goethe University Frankfurt, Theodor-W.-Adorno-Platz 4, 60629 Frankfurt, Germany; email: rahulkm85@gmail.com, Phone: +49-152-1301-0589; email: lennart.kraft@wiwi.uni-frankfurt.de; Phone +49-69-798-34769;

Table of Contents

1 Description of Methodology 3
2 Description of Data Collection 6
3 Definition of Starting and Ending Date of Monsoon 7
4 Interpretation of Coefficients for Moving Averaged Variables 7
5 Identification of Effect of Ultraviolet Index (UVI) 8
6 Model Selection to Identify the Effect of Ultraviolet Index (UVI) on the Cumulative COVID-19 Deaths 8
7 Robustness Checks 8
8 Supplementary Tables 9
9 References 16

1 Description of Methodology

We apply a Mundlak log-linear regression model to estimate the effect of the Ultraviolet Index (UVI) on the cumulative number of daily COVID-19 deaths (cumulative COVID-19 deaths). A log-linear model considers relative rather than absolute changes over time. Relative changes, such as percentage growth rates, are more comparable across different regions than absolute ones. Therefore, a log-linear model increases the comparability of across administrative regions as it considers the daily growth rates of COVID-19 deaths.

We isolate the effect of the monsoon season and UVI from region-specific time-constant factors via the Mundlak error correction term[1]. These region-specific time-constant factors include factors that are relatively stable over our observation period, such as the location (measured by its latitude and longitude) and various socio-economic characteristics of the population in a specific administrative region. This estimation strategy also controls for region-specific time-constant factors such as population density as well as age composition, skin pigmentation and rates of obesity in the population. Further, they control for healthcare status, people's lifestyle and mobility of the specific administrative region. Finally, this estimation strategy also controls for time-constant diet-related patterns in a specific administrative region such as food fortification, the composition of vegans and vegetarians as well as the consumption of dietary supplements or diets which are rich in vitamin D in the population.

The model also controls for various time-varying confounding factors via accounting for region-specific time-trends. For instance, we use the time passed by since the first reported COVID-19 infection in a specific region to control for the increasing pressure on the healthcare system over time. More importantly, this factor would also partial out any linear change in the daily growth rate of COVID-19 deaths, that is similar across regions over time. Thus, the model isolates the effect of monsoon season and UVI from an exponential-shaped

curve which is often observed in the cumulative COVID-19 deaths over time. Our model also isolates the effect of UVI from factors that influences the intensity of UVI as well as factors that affect the likelihood of exposure to Ultraviolet-B (UVB) radiation. These factors include cloud index, stratospheric ozone level, visibility level, humidity level, and temperature.

The Mundlak error correction term also captures time-constant behaviours of individuals as it captures time-constant reoccurring habits or mobility of individuals, that may affect the likelihood of exposure to UVB radiation (e.g., walking to work, outdoor exercises, outdoor activities related to work). Yet, the Mundlak error correction term does not capture time-varying factors, such as time-varying weather parameters that influence the behaviour of individuals and thus the likelihood of UVB radiation exposure.

As the onset of the monsoon season, the UVB radiation and other weather parameters plausibly affect the transmission of COVID-19 and the immunity of individuals several weeks later, we include a moving average of 56 days of the variables - monsoon season, UVI and other weather parameters in our model. We also observe that the results did not vary substantially even after increasing or decreasing these time windows used for the analysis.

We use the following model represented by equation (1) to explain the number of cumulative COVID-19 deaths:

$$D_{i,t} = D_{i,t-1} \times e^{\gamma + UVI_{i,t}\beta_{UVI} + M_{i,t}\beta_M + UVI_{i,t} \times M_{i,t}\beta_{UVI\times M} + C_{i,t}\beta_C + FI_{i,t}\beta_{FI} + u_i + \epsilon_{i,t}} \quad (1)$$

$D_{i,t}$ represents the cumulative COVID-19 deaths in an administrative region i at a point in time, t (in days). $D_{i,t}$ is related to the explanatory factors via an exponential growth model on the right-hand side of the equation (1). The exponential growth model flexibly allows for different shapes of cumulative COVID-19 deaths.

This exponential growth model consists of seven explanatory parts.

1) γ represents the daily growth rate of COVID-19 deaths from $D_{i,t-1}$ to $D_{i,t}$ that is independent the weather variables of this model. γ covers virus-specific attributes like its basic reproductive rate R_0 combined with its lethality.
2) $UVI_{i,t}$ represents the moving average of UVI for an administrative region i at day t. β_{UVI} reflects the effect of UVI.
3) $M_{i,t}$ represents the moving average of the monsoon season for an administrative region i at day t. β_M reflects the effect of M.
4) $UVI_{i,t} \times M_{i,t}$ represents the interaction of the moving average of UVI and of the moving average of M for an administrative region i at day t. $\beta_{UVI\times M}$ reflects the mitigating effect of the monsoon season on the effect of UVI.
5) $C_{i,t}$ stands for the set of control variables. This set of control variables consists of the moving average of the time-varying weather parameters such as cloud index, stratospheric ozone level, visibility level, humidity level, as well as minimum and maximum temperature for an administrative region i at day t. This set also consists of the time passed by, since the first reported COVID-19 infection for an administrative region i at day t. The vector β_C represents the effect of these control variables.
6) u_i represents time-constant region-specific factors influencing the growth rate of cumulative COVID-19 deaths (e.g., diet related effects, population parameters about their activities and demographic composition). The Mundlak error correction term isolates the effect of weather parameters such as UVI and monsoon season from this time-constant region-specific factor.
7) $\epsilon_{i,t}$ consists of all the remaining factors that are not identified but also have an effect on the cumulative COVID-19 deaths (i.e., all non-linear differences of growth rates with respect to time and region-specific linear differences of growth rates with respect to time). They could be caused by a declining number of people who could potentially

become infected or contagious, lockdowns in a region, mutation of the virus in a region over time, systematic false-reports of the dependent variable.

An appropriate transformation results in an estimable equation (2).

$$\Delta \ln(D_{i,t}) = UVI_{i,t}\beta_{UVI} + M_{i,t}\beta_M + UVI_{i,t} \times M_{i,t}\beta_{UVI\times M} + C_{i,t}\beta_C + u_i + \gamma + \epsilon_{i,t} \quad (2)$$

If γ and u_i are correlated with past or future values of the weather parameters, then the estimation of coefficients of equation (2) will be inconsistent. Therefore, we use a Mundlak model which isolates the weather variables from those time-constant factors via the error correction term. Equation (2) also shows why we can only use those observations where cumulative COVID-19 deaths are larger than zero. We present an overview of how many observations of which region we use in our analysis in Table S2.

Moreover, we acknowledge that it is possible that in a location "X", UVI may increase while the growth rate of COVID-19 deaths remains unchanged. Further, UVI may remain unchanged in another location "Y", while its growth rate of COVID-19 deaths may decrease. If such situations occur systematically, then there must be another confounding variable which causes growth rates to decrease in location Y and to remain unchanged in location X. This confounding variable would then also have an effect on the daily growth-rates of COVID-19 deaths in an analysis at a regional level, where locations X and Y are aggregated. We try to control for such confounding variables on a regional level by controlling for region-specific time-constant factors, time-varying weather parameters as well as flexible region-specific time trends.

2 Description of Data Collection

We source COVID-19 data from COVID19India.org (link: https://api.covid19india.org/documentation/csv/). We use the latitude and longitude information of administrative regions that are provided by Geocoder a geocoding library in

Python as indicated in Table S3. We collect the weather data from darksky.net based on this latitude and longitude information.

3 Definition of Starting and Ending Date of Monsoon

In order to define a variable that represents the monsoon season, we collected data concerning the onset of the monsoon season from an online newspaper article (https://indianexpress.com/article/india/from-june-2020-revised-monsoon-calendar-for-india-6364258/) and an official government website that tracks the activity of monsoon in India (https://mausam.imd.gov.in/imd_latest/contents/monsoon.php).

In order to infer the ending date of the monsoon season, we used the following procedure:

1. Calculate average probability of precipitation after the onset of the monsoon season
2. Calculate 14-days moving average of the probability of precipitation
3. Define the ending date of monsoon as the latest day, on which this 14-days moving average of the probability of precipitation drops below the overall average of the probability of precipitation after the onset of the monsoon season

We provide the starting and ending date in Table S6.

4 Interpretation of Coefficients for Moving Averaged Variables

Our structural model consists of 56 days moving averages of the previously mentioned variables. Therefore, a unit change in the monsoon season or UVI variable outlines the associated change in the daily growth-rates of COVID-19 deaths after 56 days. We define this effect as the long-run effect. A unit change in the moving average of the interaction of the monsoon season and the UVI variables outlines the mitigation of the association between the UVI and daily growth rates of COVID-19 deaths after 56 days.

5 Identification of Effect of Ultraviolet Index (UVI)

Kalippurayil Moozhipurath et al. (2020) outline the key assumption that is required to identify the causal effect of UVI[2]. The key assumption required to identify the causal effect of the monsoon season is that $M_{i,t}$ is uncorrelated to $\epsilon_{i,s}$ at all points in time, assuming that the error correction term isolates $M_{i,t}$ from all region-specific time-constant factors.

A major concern is that individuals may anticipate the monsoon season and adjust their behaviours accordingly. For instance, increased mobility just before the onset of the monsoon season may affect the likelihood of the transmission and thereby COVID-19 deaths. If such an implication is true, then the negative effect of the monsoon season on the daily growth rate of COVID-19 deaths will be downward biased. We control for such concerns by allowing for flexible time trends that isolates the effect of the monsoon season from various behavioural changes before the onset of the monsoon season. Moreover, we control for governmental measures at a national level to isolate the effect of the monsoon season from behavioural changes associated with the governmental measures.

6 Model Selection to Identify the Effect of Ultraviolet Index (UVI) on the Cumulative COVID-19 Deaths

We estimate equation (2) using various time windows ranging from 4 to 11 weeks to calculate the moving averages of the weather parameters. The results from Table S4.1 and Table S4.2 do not indicate any substantial variation concerning the size and statistical significance of the estimates of UVI, monsoon season, and their interaction. Table S4.1 and Table S4.

7 Robustness Checks

Table S5 outlines additional robustness checks and estimates the effect of the monsoon season, UVI and their interaction on the cumulative COVID-19 deaths using flexible region-specific time-trends (linear, quadratic or cubic time-trends) with governmental measures as an additional control variable. We find consistent results across different model specifications.

8 Supplementary Tables

Table S1: Correlation of weather variables

	Monsoon	UVI	Cloud	Stratospheric Ozone	Visibility	Humidity	Max Temperature	Min Tempereature
Monsoon	1							
UVI	-0.2425	1						
Cloud	0.561	-0.678	1					
Stratospheric Ozone	-0.3827	0.2962	-0.2499	1				
Visibility	-0.2886	0.2679	-0.395	0.2038	1			
Humidity	0.6176	-0.3682	0.6263	-0.4254	-0.4108	1		
Max Temperature	-0.1274	0.204	-0.1568	0.1952	0.3306	-0.3569	1	
Min Tempereature	0.2124	-0.0154	0.222	-0.0049	0.0856	0.0668	0.8559	1

Table S2: Number of observations of administrative regions used in the analysis

Administrative region	Observations	Administrative region	Observations
Andaman and Nicobar Islands	115	Ladakh	170
Andhra Pradesh	230	Madhya Pradesh	226
Arunachal Pradesh	148	Maharashtra	232
Assam	215	Manipur	113
Bihar	224	Meghalaya	202
Chandigarh	200	Mizoram	22
Chhattisgarh	174	Nagaland	118
Dadra and Nagar Haveli and Daman and Diu	135	Odisha	226
Delhi	232	Puducherry	161
Goa	150	Punjab	232
Gujarat	227	Rajasthan	229
Haryana	227	Sikkim	116
Himachal Pradesh	226	Tamil Nadu	232
Jammu and Kashmir	232	Telangana	232
Jharkhand	215	Tripura	163
Karnataka	232	Uttar Pradesh	232
Kerala	232	Uttarakhand	202
		West Bengal	229
Total Number of Observations		6,751	

Table S3: Latitude (Lat.) and longitude (Long.) of administrative regions used in analysis

Administrative region	Lat.	Long.	Administrative region	Lat.	Long.
Andaman and Nicobar Islands	10.22	92.58	Ladakh	33.95	77.66
Andhra Pradesh	15.92	80.19	Madhya Pradesh	23.97	79.39
Arunachal Pradesh	27.69	96.46	Maharashtra	19.53	76.06
Assam	26.41	93.26	Manipur	24.72	93.92
Bihar	25.64	85.91	Meghalaya	25.54	91.30
Chandigarh	30.73	76.78	Mizoram	23.21	92.87
Chhattisgarh	21.66	81.84	Nagaland	26.16	94.59
Dadra and Nagar Haveli and Daman and Diu	20.72	70.93	Odisha	20.54	84.69
Delhi	28.65	77.22	Puducherry	11.93	79.83
Goa	15.30	74.09	Punjab	30.93	75.50
Gujarat	22.39	71.75	Rajasthan	26.81	73.77
Haryana	29.00	76.00	Sikkim	27.60	88.45
Himachal Pradesh	31.82	77.35	Tamil Nadu	10.91	78.37
Jammu and Kashmir	33.56	75.06	Telangana	17.85	79.12
Jharkhand	23.46	85.26	Tripura	23.78	91.70
Karnataka	14.52	75.72	Uttar Pradesh	27.13	80.86
Kerala	10.35	76.51	Uttarakhand	30.09	79.32
			West Bengal	23.00	87.69

Table S4.1: Effect of the monsoon season, Ultraviolet Index (UVI) and their interaction on cumulative COVID-19 deaths (robustness checks)

	Model A.1	Model A.2	Model A.3	Model A.4
Dependent Variable	COVID-19 Deaths	COVID-19 Deaths	COVID-19 Deaths	COVID-19 Deaths
UVI	-0.001<br>(-0.24)	-0.002<br>(-0.51)	-0.004<br>(-0.96)	-0.009*<br>(-2.13)
Monsoon	-0.008<br>(-0.35)	-0.028<br>(-1.09)	-0.040<br>(-1.44)	-0.050+<br>(-1.68)
UVI x Monsoon	0.003<br>(1.03)	0.005+<br>(1.83)	0.006*<br>(2.06)	0.007*<br>(2.14)

Control variables

Time Windows used for Moving Average in Days	28	35	42	49
Time Trend of Growth Rate	Linear and Square (region-specific)	Linear and Square (region-specific)	Linear and Square (region-specific)	Linear and Square (region-specific)
Cloud Index	Yes	Yes	Yes	Yes
Stratospheric Ozone Level	Yes	Yes	Yes	Yes
Visibility Level	Yes	Yes	Yes	Yes
Humidity Level	Yes	Yes	Yes	Yes
Temperature (min and max)	Yes	Yes	Yes	Yes
Number of Estimates	9 (+9 MEC + 70 RSTE)	9 (+9 MEC + 70 RSTE)	9 (+9 MEC + 70 RSTE)	9 (+9 MEC + 70 RSTE)
Number of Observations	6,751	6,751	6,751	6,751
Number of regions	35	35	35	35
R-squared Within	10.20%	10.29%	10.32%	10.30%

Note: +: p < 0.10, *: p < 0.05, **: p < 0.01. t-statistics to test statistical significance (two-sided test) of the estimated associations between the dependent variable and the respective independent variable in parentheses. MEC represents Mundlak error correction estimates which isolate the weather variables from region-specific time-constant factors. RSTE represents region-specific time-effects.

Table S4.2: Effect of the monsoon season, Ultraviolet Index (UVI) and their interaction on cumulative COVID-19 deaths (robustness checks)

	Model A.5	Model A.6	Model A.7	Model A.8
Dependent Variable	COVID-19 Deaths	COVID-19 Deaths	COVID-19 Deaths	COVID-19 Deaths
UVI	-0.012** (-2.64)	-0.012* (-2.34)	-0.012* (-2.32)	-0.014* (-2.46)
Monsoon	-0.075* (-2.33)	-0.113*** (-3.32)	-0.117*** (-3.33)	-0.135*** (-3.79)
UVI x Monsoon	0.009* (2.56)	0.013*** (3.34)	0.013*** (3.40)	0.014*** (3.59)

Control variables

Time Windows used for Moving Average in Days	56	63	70	77
Time Trend of Growth Rate	Linear and Square (region-specific)	Linear and Square (region-specific)	Linear and Square (region-specific)	Linear and Square (region-specific)
Cloud Index	Yes	Yes	Yes	Yes
Stratospheric Ozone Level	Yes	Yes	Yes	Yes
Visibility Level	Yes	Yes	Yes	Yes
Humidity Level	Yes	Yes	Yes	Yes
Temperature (min and max)	Yes	Yes	Yes	Yes
Number of Estimates	9 (+9 MEC + 70 RSTE)	9 (+9 MEC + 70 RSTE)	9 (+9 MEC + 70 RSTE)	9 (+9 MEC + 70 RSTE)
Number of Observations	6,751	6,751	6,751	6,751
Number of regions	35	35	35	35
R-squared Within	10.34%	10.45%	10.43%	10.38%

Note: +: p < 0.10, *: p < 0.05, **: p < 0.01. t-statistics to test statistical significance (two-sided test) of the estimated associations between the dependent variable and the respective independent variable in parentheses. MEC represents Mundlak error correction estimates which isolate the weather variables from region-specific time-constant factors. RSTE represents region-specific time-effects.

Table S5: Effect of the monsoon season, Ultraviolet Index (UVI) and their interaction on cumulative COVID-19 deaths (flexible time trend & governmental measures)

	Model B.1	Model B.2
Dependent Variable	COVID-19 Deaths	COVID-19 Deaths
UVI	-0.14*<br>(-2.31)	-0.0059<br>(-0.85)
Monsoon	-0.075*<br>(-2.08)	-0.075+<br>(-1.74)
UVI x Monsoon	0.0083*<br>(2.01)	0.011*<br>(2.43)

Control variables

Time Trend of Growth Rate	Linear, Square and Cubic (region-specific)	Linear, Square and Cubic (region-specific)
Governmental Measures	No	Yes
Cloud Index	Yes	Yes
Stratospheric Ozone Level	Yes	Yes
Visibility Level	Yes	Yes
Humidity Level	Yes	Yes
Temperature (min and max)	Yes	Yes
Number of Estimates	9 (+9 MEC + 105 RSTE)	17 (+17 MEC + 105 RSTE)
Number of Observations	6,751	6,751
Number of Regions	35	35
R-squared Within	12.71%	14.44%

Note: +: $p < 0.10$, *: $p < 0.05$, **: $p < 0.01$. t-statistics to test statistical significance (two-sided test) of the estimated associations between the dependent variable and the respective independent variable in parentheses. MEC represents Mundlak error correction estimates which isolate the weather variables from region-specific time-constant factors. RSTE represents region-specific time-effects.

Table S6: Number of observations of administrative regions used in analysis

Administrative region	Monsoon Start	Monsoon End	Administrative region	Monsoon Start	Monsoon End
Andaman and Nicobar Islands	22.05.2020	05.11.2020	Ladakh	23.06.2020	19.09.2020
Andhra Pradesh	11.06.2020	26.10.2020	Madhya Pradesh	21.06.2020	29.09.2020
Arunachal Pradesh	05.06.2020	09.10.2020	Maharashtra	11.06.2020	26.10.2020
Assam	04.06.2020	11.10.2020	Manipur	05.06.2020	10.10.2020
Bihar	18.06.2020	09.10.2020	Meghalaya	05.06.2020	10.10.2020
Chandigarh	26.06.2020	12.09.2020	Mizoram	04.06.2020	11.10.2020
Chhattisgarh	16.06.2020	21.10.2020	Nagaland	13.06.2020	13.10.2020
Dadra and Nagar Haveli and Daman and Diu	24.06.2020	28.09.2020	Odisha	12.06.2020	19.10.2020
Delhi	27.06.2020	13.09.2020	Puducherry	01.06.2020	01.11.2020
Goa	07.06.2020	26.10.2020	Punjab	28.06.2020	15.09.2020
Gujarat	24.06.2020	01.10.2020	Rajasthan	03.07.2020	16.09.2020
Haryana	01.07.2020	15.09.2020	Sikkim	11.06.2020	08.10.2020
Himachal Pradesh	24.06.2020	17.09.2020	Tamil Nadu	04.06.2020	23.09.2020
Jammu and Kashmir	23.06.2020	16.09.2020	Telangana	08.06.2020	25.10.2020
Jharkhand	14.06.2020	16.10.2020	Tripura	04.06.2020	10.10.2020
Karnataka	05.06.2020	28.10.2020	Uttar Pradesh	22.06.2020	11.09.2020
Kerala	01.06.2020	24.10.2020	Uttarakhand	20.06.2020	20.09.2020
			West Bengal	08.06.2020	15.10.2020

9 References

1. Mundlak, Y. On the pooling of time series and cross section data. *Econom. J. Econom. Soc.* 69–85 (1978).
2. Moozhipurath, R. K., Kraft, L. & Skiera, B. Evidence of protective role of ultraviolet-B (UVB) radiation in reducing COVID-19 deaths. *Sci. Rep.* **10**, 17705 (2020).
